# Agonal Thrombus at Necropsy—A Third Category of Blood Coagulation in Domestic Carnivores

**DOI:** 10.3390/life13091834

**Published:** 2023-08-30

**Authors:** Raluca Elena Tiu, Raluca Ioana Rizac, George Laurentiu Nicolae, Raluca Mihaela Turbatu, Emilia Ciobotaru-Pirvu

**Affiliations:** Faculty of Veterinary Medicine, University of Agronomic Sciences and Veterinary Medicine of Bucharest, 011464 Bucharest, Romania

**Keywords:** thrombus, agonal thrombus, agonal death, necropsy, veterinary forensic pathology, cruor, sudden death

## Abstract

Agonal thrombus is infrequently discussed in veterinary forensic pathology, being misdiagnosed as postmortem coagulation. The main purpose of the present study is to confirm that agonal thrombosis is an important tool in ruling out sudden death and to characterize it by gross, histological, and immunohistochemical approaches. The investigations have been conducted on 56 domestic carnivores. Fibrin was observed as rosette—like arrangements around platelet aggregates, loose network, wave—like pattern or short fibers and the additional tendency of lines of Zahn being noted inconsistently. All agonal thrombi had positive reactions for anti—CD61 for platelets, disposed in variable—sized clumps or in a linear pattern close to endothelial cells of endocardium. The same positive reaction has been noted to anti—fibrinogen and anti—fibrin antibodies. CD45, CD68 and von Willebrand factor had a very low to absent expression. Cardiac lesions were found in 22 cases (39.29%) suggesting predisposition to agonal thrombosis in animals with cardiovascular diseases. The results prove that agonal thrombus is a third category of blood coagulation that forms strictly during agonal death. The microscopical findings describe the agonal thrombus similar to the morphology of recent thrombus vera. Given the results, the agonal thrombus is a useful tool that confirms the agonal suffering prior to death.

## 1. Introduction

It is well known that the intra—vitae coagulation process is divided into thrombosis which occurs in the lumen of the vessels and clotting which refers to the extravascular process with the role to repair the damaged tissue [1,2]. After death onset, the coagulation process that takes place naturally in a body is called postmortem clotting and is defined by the identification of cruors during necropsy [3]. Given the importance of knowledge in either treatment and forensic pathology, the differentiation between antemortem and agonal thrombosis, and postmortem coagulation needs further clarification and study, being of paramount importance when all three types or their combinations may be identified in the same individual. Of note, the early stages of thrombus formation should be subjected to differentiation when overlapped antemortem, agonal, and postmortem changes occur.

The outcomes of research in the field on blood coagulation, the understanding of the mechanisms and the improvement of treatment methods in thromboembolic diseases in both human and animals have recorded huge steps. In the mechanisms of thrombus formation, three conditions are of major importance (Virchow’s triad) [4,5]. Vessel wall injuries or endothelial alterations alongside changes in the biochemical profile of blood toward hypercoagulability and impairment of blood flow in the cardiovascular system are the elements that contribute to thrombus formation [6].

The thrombus is formed via an intrinsic or extrinsic pathway leading to the formation of a stable structure attached to the blood vessel mainly formed out of fibrin, platelets and blood cells, usually presenting a laminated pattern, known as lines of Zahn [7,8]. It is also highlighted in previous studies that the incidence of lines of Zahn is higher in heart and aorta thrombosis and smaller in veins and small arteries [9]. The lines of Zahn had been considered pathognomonic for antemortem thrombus, being characterized by many definitions, all of them referring to layered distribution of platelets, fibrin and erythrocytes [10]. Comparatively, agonal thrombi may also present these features, the layering of fibrin and blood cells being observed to a certain extent [11].

For improving the recognition capacity of thrombus components, immunohistochemistry can provide additional results to classic histopathology. Thus, the estimation of age is based on the prevalence of platelets, fibrin and cellular infiltration [12].

Depending on the age of the thrombus, these elements are expressed differently: in early thrombus (1 h) platelets, factor VIII, and fibrinogen are observed with no infiltration of lymphocytes; in recent thrombus (1–24 h) the lymphocytes are observed, recent—medium thrombus (24–48 h) shows a high positive reaction to lymphocytes progressing to a medium—aged thrombus (48–72 h) with the presence of fibroblasts; over 72 h (old thrombus) the process of recanalization and fibrosis are the main characteristics [13]. For more elements of differentiation between postmortem clotting and antemortem thrombus, serpiginous bands of nested platelets and fibrin associated with extended neutrophil karyorrhexis have been considered the most reliable features [14]. By comparison, the red or black cruors (post—mortem clots) are just masses of sedimented red blood cells with few leukocytes [11,15,16]. 

The agonal thrombus is a structure formed strictly in articulo—mortis, associated with cases of longtime agonal suffering. Studies performed in humans proved that agonal thrombi have not been identified in sudden death cases [11,17,18]. Agonal thrombus is described in the literature as having a soft to mild consistency with a finely granular surface and little to no adherence to the endothelium, while the microscopic findings described some layering of blood components, resembling lines of Zahn [11,15]. 

Forensic veterinary pathology is currently facing numerous cases of cruelty or abuse, most of the victims being domestic dogs and cats. As a result of this pressure, supplementary clarifications are needed in order to properly document whether sudden death or prolonged agony and suffering occurred. Even though agonal thrombus is not significant for the cause of death, its presence has an important role, confirming that the animal suffered an agonal period of time prior to death. There are several studies in humans that warn regarding the risk of considering agonal thrombi as true clots (also called in other research papers “chicken fat” clot) [19]. Thus, gross examination could lead to misinterpretation, especially when agonal thrombosis continues after death with the formation of postmortem cruors. In order to accurately differentiate the aforementioned structures, gross examination associated with histological and immunohistochemical features is being considered.

The research articles about the agonal thrombus have no characterization of the immunohistochemical aspects, except the gross and standard microscopic examination. The main purpose of the present study is to characterize the agonal thrombus in domestic carnivores, giving the forensic veterinary pathologist a standard method to rule out sudden deaths in these species.

## 2. Materials and Methods

The cases of agonal suffering included in the study (*n* = 56) have been represented by 26 dogs (46.43%) and 30 cats (53.57%) of different breeds, ages and body weight. The caseload has been selected from the teaching hospital of University of Agronomic Sciences and Veterinary Medicine Bucharest. Agonal suffering has been diagnosed for an extensive time in all cases (Table 1 and Table 2), being based on the clinical findings such as hypotension, hypothermia, respiratory distress, loss of consciousness, lethargy and profound weakness. Additionally, one case of sudden death in a dog with dilated cardiomyopathy and one case of aortic thromboembolism in a cat have been considered as the reference in order to make a comparative study between cruors, agonal thrombi, and antemortem thrombosis (Table 2).

Standard necropsy has been performed (within 2–12 h post—mortem on fresh tissue) followed by sampling of the agonal thrombus from the right atrium and ventricle of the heart alongside the myocardial tissue, cruors, aortic and renal artery thrombus. The samples were fixed in 10% buffered formaldehyde, trimmed, embedded in paraffin, and sectioned into 5.0 μm sections for hematoxylin—eosin staining (H&E), Mallory and Masson Trichrome for fibrin staining.

For immunohistochemistry staining, antibodies against CD45 (lymphocytes), CD61 (platelets), CD68 (monocytes/macrophages), von Willebrand factor, fibrinogen, and anti—fibrin (D—dimer fibrin) were used. Following the protocol, the samples were deparaffinized and rehydrated by a series of xylen and alcohol washes, followed by heat—induced antigen retrieval, washed in tris—buffered—saline with Tween 20 (TBS Tween 20). Primary antibody incubation with antibody dilution 1:300 for 24 h at 4 °C was followed by secondary antibody incubation with chromogenic marker. Nuclear counterstaining was used, providing better context and contrast for the antibodies. 

## 3. Results

### 3.1. Gross Examination 

**Figure 1:**
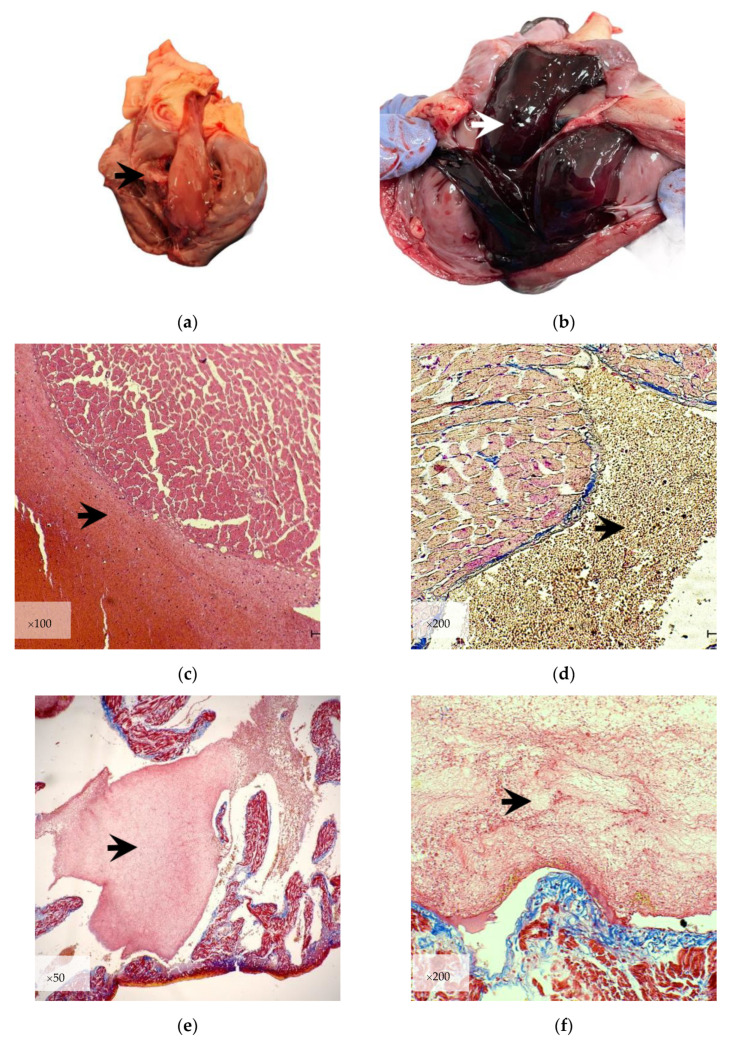
(**a**) Gross examination of agonal thrombus in dog (right heart chambers) depicting so called “chicken fat” clot; notice the agonal thrombus intertwining cordinae tendinae; (**b**) gross examination of postmortem black `currant jelly cruor in dog with sudden death (white arrow); (**c**) microscopical examination in cruors H&E ×100—notice the compact erythrocytes mass with no fibrin layering; (**d**) microscopical examination of red cruors Mallory Trichrome staining ×200—notice the mass of erythrocytes with no fibrin expression; (**e**) microscopical examination of agonal thrombi Masson Trichrome staining ×50—red stained fibrin block within the agonal thrombus; (**f**) agonal thrombi: red stained wave—like fibrin (black arrow) Masson Trichrome staining ×200.

The presence of agonal thrombi especially in the right heart chambers was confirmed (Figure 1a). They all had a soft—mild consistency, variable in color depending on the blood sedimentation. All the agonal thrombi had a discontinuous mild adherence at the endocardium. In addition, most of the cases had the agonal thrombi intertwined among the chordae tendineae, proving that the heart was beating during the formation of agonal thrombi. Post—mortem coagulation cruors were non—adherent to the endocardium with a soft consistency, dark red or black in color with a glossy surface—as previously described “black currant jelly” cruors (Figure 1b). Table 3 present comparatively the features of red cruors, aortic thrombus and agonal thrombi.

### 3.2. Microscopic Examination

#### 3.2.1. Histological Evaluation

The microscopical findings of the agonal thrombus were similar in all the cases included in the present study. Discontinuous, patch—like adherence to the endothelial layer of the endocardium was generally observed. The mass of agonal thrombus consists of fibrin and blood cells, frequently arranged in layers. Sometimes in small veins of the heart, agonal thrombi clearly expressed clusters of leukocytes wrapped in fine layers of fibrin. The disposition of fibrin has been equally observed as waves intertwining between chordae tendineae or as a fine, loose network with scattered red blood cells or leukocytes (Figure 1e,f).

Compared with the agonal thrombi, cruors do not have fibrin deposition, being mainly formed out of blood cells, especially erythrocytes (Figure 1c,d)

**Scheme 1:**
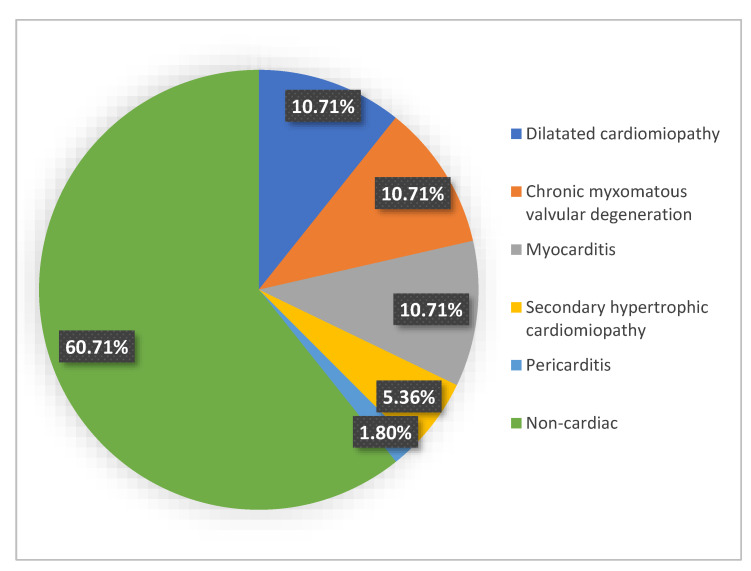
Illustrating cardiac lesion distribution associated with agonal thrombosis.

Histological evaluation revealed the cardiac lesions found in 22 cases (39.29%) were represented by chronic myxomatous valvular degeneration corresponding to mitral valve disease (10.71%), cardiomyocyte degeneration consistent with dilated cardiomyopathy (10.71%) and myocarditis (10.71%) (Table 2 and Scheme 1). The remaining 34 cases (60.71%) suffered a non—cardiac death such as: trauma (14.28%), infectious diseases (12.50%) or tumoral disease (8.92%) (Table 1). 

Mallory and Masson trichrome staining clearly captured more substantial deposition of fibrin, such as amorphous blocks or wave—like arranged, suggesting the formation of lines of Zahn (Figure 1e,f). This feature was highly specific considering the formation of agonal thrombus started ante—mortem. The red stained fibrin of agonal thrombus was associated with scattered leukocytes and erythrocytes. 

#### 3.2.2. Immunohistochemistry

**Figure 2:**
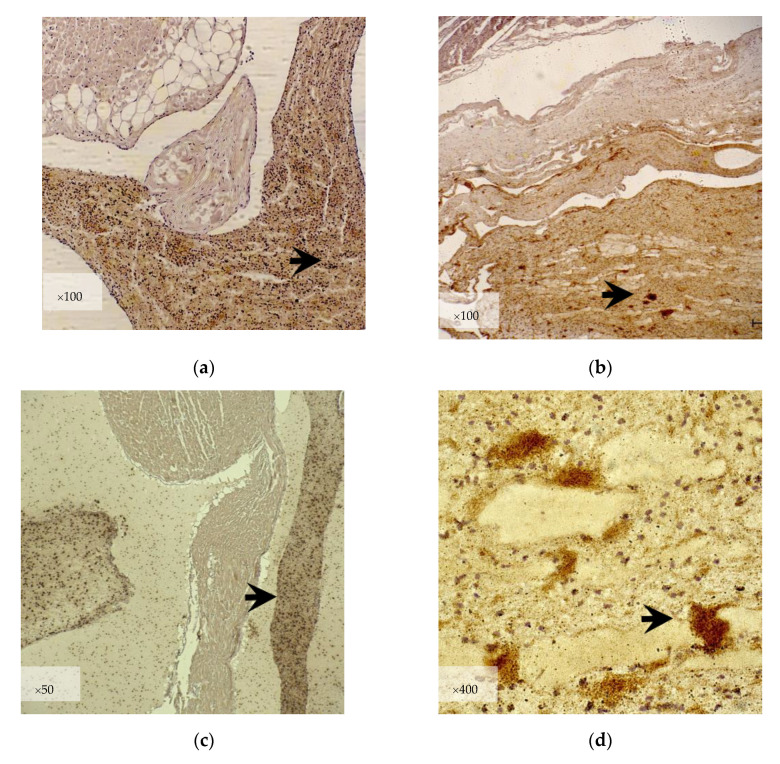

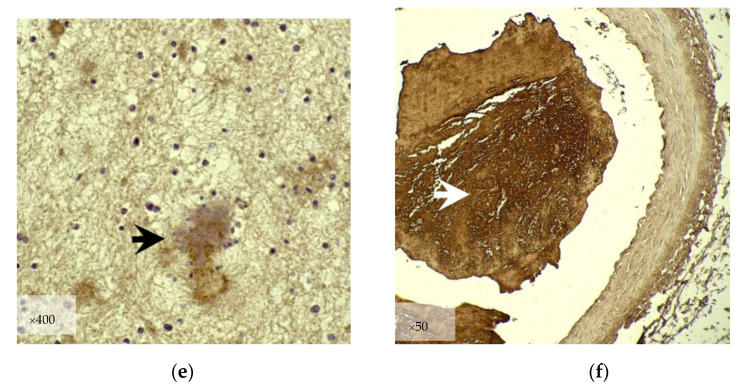
(**a**) Immunohistochemistry—anti—fibrinogen labeling presenting strong positive reaction bond to red blood cell mass (black arrow), ×100; (**b**) anti—platelets staining (CD61)—strong positive reaction arranged in clumps or laminated pattern (black arrow), ×100; (**c**) anti—fibrin (d—dimer) staining × 50—laminated disposition resembling to lines of Zahn (arrow); (**d**) anti—platelet staining CD—61, clumped platelets, ×400; (**e**) meshwork and rosette—arranged strands of fibrin attached to the clumped platelets and nuclear fragments (black arrow); leukocytes (neutrophils are dominant) are entrapped in the fibrin meshwork, ×400; (**f**) fibrin staining in renal artery thromboembolism presenting strong positive reaction and diffuse, compact pattern (white arrow), ×50.

All the agonal thrombi were mostly constituted of platelets (CD61—anti—platelets) and fibrin. The anti CD61 showed strong positivity in all selected cases, arranged in variable sized clumps or linear disposition mostly alongside the cardiac endothelial cells or within the thrombus mass (Figure 2b). Fibrinogen showed a positive reaction, mainly bound to the erythrocytes in the middle layers of the agonal thrombus (Figure 2a). Fibrin immunostaining showed a strong positive reaction within the entire body of the agonal thrombus. Fibrin labeling was heterogeneous, as a very fine network or short fibers attached in a rosette—like pattern to small clumps of platelets or as a wave—like pattern (Figure 2c–e). The leukocytes, especially neutrophils, were clumped within a fibrin meshwork presenting normal cellular features or inconsistent nucleus fragmentation (karyorrhexis). 

Immunohistochemistry labeling of fibrin was also used in a case of a cat aortic thromboembolism and subsequent renal artery thromboembolism. In thromboemboli, fibrin was consistent with stronger staining indicating a highly packed meshwork of fibrin (Figure 2f) [20].

CD45 (anti—lymphocytes), CD68 (anti—monocytes/macrophages) and von Willebrand factor showed an absent or a very low positive reaction within the agonal thrombus (Table 4). Comparative aspects between agonal thrombus and antemortem thrombus regarding the immunohistochemical features are presented in Table 5.

## 4. Discussion

Blood coagulation is a very complex process which raises important questions regarding hemostasis, thrombosis and postmortem clotting. Considering the situations which can undoubtedly overlap during necropsy and histopathological diagnosis, the pathologist should always consider a solid morphological differentiation.

Early blood antemortem coagulation is represented by the activation of zymogens of serine proteases by limited proteolytic cleavage. The outcome of this process is procoagulant cascade, converting prothrombin in thrombin. The latter divides in four peptides, bonding subsequently with soluble fibrinogen and resulting insoluble fibrin. Coagulation process requires mandatory phospholipidic surfaces provided by adherent, clustered activated platelets to the place of tissue or vascular injury. Activation of platelets is mainly induced via subendothelial collagen or by vessel wall or blood—borne tissue factor. This second provenience of tissue factor does not request disruption of the endothelial cells and is independent from von Willebrand factor. This will engage changes in surface lipid composition, releasing of platelet procoagulant factors from corresponding granules and formation of a more robust, stable thrombus, due to a highly branched fibrin meshwork [20,21]. 

Thrombosis and thromboembolism in veterinary medicine are still underrated conditions, diagnostic—wise and treatment—wise. Prothrombotic conditions in domestic carnivores include sepsis, neoplasia, heartworm disease, trauma, heart disease, hyperadrenocorticism, immune—mediated hemolytic anemia, and protein—losing nephropathy. Therefore, in depth research is needed in order to clarify the morphology of antemortem thrombi, as well as to differentiate them from agonal thrombi and cruors [21,22].

Publications regarding agonal thrombosis in animals present little information to veterinary pathologists, being represented by preliminary studies concerning the histological features of these structures [17,18,23]. The present study aimed to characterize the agonal thrombi compared to cruors and thrombus vera.

The gross examination of cruors revealed similar features to those mentioned in the literature: soft consistency with a glossy surface, usually red—dark or black colored and with no adherences to the endocardium [14,15,17,18]. Comparatively, agonal thrombus is finely attached to endocardium, yellow to red color with a soft to mild consistency and smooth surface. Based on our practical experience, this discrete adherence to smooth endocardial surface is being kept until postmortem hemolysis. Further studies are needed in order to understand the relationship between agonal thrombus and endocardium by using electron microscopy.

Special staining for fibrin and immunohistochemistry presented strong positive reaction for fibrin and platelets, being the most important features of agonal thrombus. Aggregation on platelets was highly encountered. This study cannot present evidence regarding the activation of platelets independent from other antemortem conditions which can create a prothrombotic state. Given the cause of death in cases considered for the study, it can be observed that preconditions of platelet activation and subsequent aggregation have been created in several diseases. In our study, five cases of heartworm have been associated with agonal thrombosis. Although notable thrombus formation has not been observed while parasites are alive, platelet hyperactivity has been reflected in the tendency to aggregate into the filarial surface. Moreover, the whole body extract of adult male of *Dirofilaria immitis* creates a thromboembolic state, which is generated by adult metabolic products or by antigen—derived molecules of *Wolbachia* spp. [24]. Still, our results proved a platelet—platelet affinity, which subsequently entails fibrin adherence.

As our results proved, the most important features which differentiate agonal thrombi from cruors are the platelets aggregation associated with fibrin deposition. The layering of fibrin within agonal thrombus formation is a time—influenced process, being usually associated with the formation of a fine meshwork or laminated lines resembling lines of Zahn. This feature is unanimously accepted as specific for true thrombus, when fibrin arrangements present a geographic pattern alongside highly packed platelets [13,25,26]. Layering of the fibrin deposition of the agonal thrombus proves that their formation starts during the lifetime of the animal, especially in agonal suffering. It is also noteworthy that within lines of Zahn, the relationship between platelets and fibrin has been proven by an observed rosette—like arrangement. This manner of arrangement is different from true thrombi which present a more robust fibrin and platelet representation [15].

The formation of the agonal thrombus is associated with agonal suffering that is caused by any disease. The agonal suffering is a state that progressively leads to change in the blood flow, slowly shutting down the cellular and organic functions. One theory suggests that the lost/low motility of the thoracic cavity could play a role, presuming that cardiac dysfunction and output failure of the heart determinates an alteration of blood flow that could be the cause for agonal thrombi formation [19,24,25,26,27]. Alongside the presence of fibrin in the structure of the agonal thrombus, fine adherences to the cardiac endothelial surface were observed. Thus, it can be suggested that the hypoxia—induced injuries of endothelial cell could play a major role in the formation of agonal thrombosis due to the expression and function of endothelial integrin receptors, initiating platelet adherence [28,29]. This present study does not describe any endothelial cell lesions, nor exposure of subendothelial collagen, known as being one of the most important conditions for initiating thrombus formation. The agonal changes of endothelial cells of the endocardium could play a role in agonal thrombus formation; future studies in this area are being considered by the authors.

Comparatively, red or black cruors are represented by solid mass of erythrocytes and white blood cells. The results of immunohistochemistry did not identify aggregation of platelets and fibrin deposition, proving that CD61 and anti—fibrin antibodies are useful tools to differentiate cruors from agonal thrombi.

Differentiation of agonal thrombi from thrombus vera may sometimes be a very challenging task, because thrombus formation is a dynamic process, which is subjected to continuous changes produced by come—and—go platelets, fibrin formation and recruitment of cells from bloodstream and vascular wall. As presented in anterior studies regarding the age of the antemortem thrombi, significant resemblances have been noted between the components of agonal thrombi and antemortem thrombi in early stages, the latter being also formed by platelets, fibrinogen and fibrin [3,7,8,13,27,30,31,32].

Immunohistochemical examination highlighted some common features, such as a strong reaction of platelets in the vicinity of the endothelium and platelet aggregation. Given the fact that platelets are structures that are being recruited earlier, in correlation with the lack of mononuclear cells in the body of the agonal thrombus, this proves that the agonal thrombosis is initiated shortly before death as it is presented in early antemortem thrombus.

The mononuclear cells found in the agonal thrombi showed little to absent immunostaining, as expected, knowing the lymphocytes and macrophages are a late—marker for a chronic reaction. The absence of blood—derived neutrophils, lymphocytes and monocytes/macrophages proves that the agonal thrombus does not express features which are a hallmark for the thrombus resolution [33].

Von Willebrand factor has presented absent or very low labeling in agonal thrombus. This glycoprotein is one of the major coagulation factors usually found in endothelial cells or megakaryocytes. It is involved in the formation of thrombus vera maintaining platelets cohesion to the injured site. Conventionally, von Willebrand factor mediates at the site of vascular injury the platelets adhesion and the formation of platelet aggregations. In order for it to activate the chain of thrombosis, subendothelial perturbation needs to occur. These conventional mechanisms have been extended to include independent activation of platelet aggregation [29]. This mechanism of independent activation and adherence of platelets has been demonstrated by initiating the thrombus formation modulated by activated platelets membrane glycoprotein α_IIb_β_3_. Via this pathway, platelet aggregation forms by binding with fibrinogen to α_IIb_β_3_ on another platelet [34]. A study shows that under shear rate stress induced in mice, the activation—independent platelet aggregation is the possible initiation of thrombus formation, before von Willebrand factor activation [29,30,31,32,33,34,35].

Absent reaction to von Willebrand factor within the agonal thrombus demonstrates no contribution to agonal thrombus formation, due to lack of subendothelial collagen exposure. In the absence of von Willebrand factor implication, blood—borne tissue factor might be considered as the second initiator of platelet aggregation in agonal thrombosis [20].

Based on the current result, some areas remain worth investigating in the future research, namely the prevalence of agonal thrombosis in the domestic carnivores population, the postmortem degradation of agonal thrombus according to environmental conditions and the relationship between agonal thrombus and endothelial cells of the endocardium. Furthermore, there are other mechanisms to be considered that could trigger the formation of agonal thrombosis: dysregulation of hemostasis associated with the dying process that can induce activation of coagulation factors and platelet activity during the terminal stage of life; hypoxia and ischemia that frequently accompany the agonal suffering, leading to oxygen deficiency and the activation of procoagulant factors [36].

## 5. Conclusions

Agonal thrombosis is complex phenomenon that engages major steps of antemortem coagulation: platelet aggregation and fibrin meshwork formation. Understanding its mechanisms and forensic significance can provide valuable insights into the hemostatic changes occurring during agonal suffering. 

This is the first study that describes the agonal thrombus using immunohistochemistry in domestic carnivores. Antemortem thrombi, postmortem clots and agonal thrombi are three important clots/thrombi found during necropsy that need to be described separately, given their major importance in the forensic pathology, especially when death of the animal occurs in unknown or obscure circumstances. Given the presence of fibrin and platelets, it can be easily confirmed that agonal thrombosis is a third category of thrombosis that forms during articulo mortis. 

Compared with a recent antemortem thrombus, it seems that the agonal thrombi are alike, having the morphological and microscopic particularities of an early—staged thrombus. It is important to use the features of agonal thrombi in order to properly rule out sudden death scenarios. 

## Figures and Tables

**Table 1:** Necropsy cases without cardiac lesions associated with agonal thrombus.

Species	Breed	Age	Gender	Cardiac Lesions	Cause of Death
Canine	Golden Retriever	1 year old	M	No	Congenital renal dysplasia
Canine	Half breed	14 years old	F	No	Metastazised mammary carcinoma
Canine	Half breed	12 years old	F	No	Peritonitis
Canine	Siberian Husky	10 years old	M	No	Splenic torsion
Canine	Presa Canario	6 months old	F	No	Gastric and splenic torsion
Canine	Half breed	4 months old	F	No	Purulent meningoencephalitis
Canine	Half breed	13 years old	M	No	Gastric torsion
Canine	Half breed	10 years old	F	No	Metastazised mammary carcinoma
Canine	Half breed	9 months old	F	No	Canine Parvovirus
Canine	Half breed	5 years old	M	No	Pulmonary edema
Canine	French Bulldog	5 years old	F	No	Traumatic subdural hematoma
Canine	Half breed	8 years old	F	No	Metastazised mammary carcinoma
Feline	Domestic shorthair	2 years old	M	No	Pulmonary edema
Feline	Domestic shorthair	2 years old	M	No	Feline Coronavirus
Feline	Domestic shorthair	3 months old	M	No	Pulmonary edema
Feline	Persian	9 months old	M	No	Pulmonary edema
Feline	Main Coon	6 months old	F	No	Feline panleukopenia virus
Feline	Domestic shorthair	7 years old	M	No	Feline panleukopenia virus
Feline	Domestic shorthair	7 years old	M	No	Pulmonary edema
Feline	Main Coon	6 months old	F	No	Feline panleukopenia virus
Feline	Domestic shorthair	6 years old	F	No	Feline Coronavirus
Feline	Domestic shorthair	3 years old	M	No	Stercoral peritonitis
Feline	Domestic shorthair	2 years old	M	No	Feline Coronavirus
Feline	Domestic shorthair	3 years old	M	No	Traumatic— trans—diaphragmatic hernia
Feline	Domestic shorthair	5 years old	M	No	Traumatic hepatic rupture
Feline	Domestic shorthair	4 years old	M	No	Traumatic trans—diaphragmatic hernia
Feline	Domestic shorthair	4 years old	M	No	Esophagian rupture
Feline	Domestic shorthair	2 years old	F	No	Pulmonary edema
Feline	Domestic shorthair	6 months old	F	No	Traumatic hepatic rupture
Feline	Ragdoll	4 months old	M	No	Feline panleukopenia virus
Feline	Domestic shorthair	10 years old	F	No	Metastazised osteosarcoma
Feline	Domestic shorthair	10 years old	F	No	Metastazised mammary carcinoma
Feline	Domestic shorthair	1 year old	F	No	Idiopathic chilothorax
Feline	Domestic shorthair	5 years old	F	No	Traumatic— trans—diaphragmatic hernia

**Table 2:** Necropsy cases with cardiac lesions associated with agonal thrombus.

Species	Breed	Age	Gender	Cardiac Lesions	Cause of Death
Feline	Domestic shorthair	10 years old	F	Yes	Secondary hypertrophic cardiomyopathy
Feline	Domestic shorthair	10 years old	F	Yes	Secondary hypertrophic cardiomyopathy
Canine	Half breed	10 years old	F	Yes	Dirofilariasis
Canine	Half breed	Adult	M	Yes	Dirofilariasis
Canine	Half breed	14 years old	F	Yes	Dirofilariasis
Canine	Half breed	5 years old	M	Yes	Dirofilariasis
Canine	Half breed	8 years old	M	Yes	Mitral valve disease
Canine	Half breed	8 years old	M	Yes	Dirofilariasis
Canine	French Bulldog	5 years old	F	Yes	Mitral valve disease
Canine	Half breed	Adult	M	Yes	Mitral valve disease
Canine	Half breed	12 years old	F	Yes	Mitral valve disease
Canine	German Shepard	5 years old	M	Yes	Dirofilariasis
Canine	Half breed	12 years old	M	Yes	Mitral valve disease
Canine	Bullmastiff	6 years old	F	Yes	Acute myocarditis
Canine	German Shepard	1 month old	F	Yes	Acute myocarditis— Canine Parvovirus
Canine	Half breed	14 years old	F	Yes	Mitral valve disease
Feline	Domestic shorthair	3 years old	M	Yes	Secondary hypertrophic cardiomyopathy
Feline	Domestic shorthair	5 years old	F	Yes	Acute myocarditis
Feline	Domestic shorthair	5 years old	M	Yes	Myocarditis
Feline	Domestic shorthair	2 months old	F	Yes	Myocarditis
Feline	Domestic shorthair	6 years old	F	Yes	Acute myocarditis
Feline	Domestic shorthair	2 years old	F	Yes	Pericarditis
Feline *	Domestic shorthair	9 years old	F	Yes	Aortic thromboembolism
Canine **	Half breed	13 years old	M	Yes	Dilated cardiomyopathy

* reference case for antemortem thrombosis; ** reference case for postmortem coagulation (black cruors).

**Table 3:** Comparative gross features of thrombi, cruors and agonic thrombi.

	Thrombi	Cruors	Agonal Thrombi
Consistency	Firm to crumble	Soft	Soft to mild
Color	Variable	Dark red, black colored	Variable
Adherence	STRONG adherence	NO adherence	MILD adherence

**Table 4:** Immunohistochemical markers for agonal thrombus.

	Low to Absent Reaction	Positive Reaction	Strong Reaction
CD45	Yes	No	No
CD61	No	Yes	Yes
CD68	Yes	No	No
Von Willebrand factor	Yes	No	No
Fibrinogen	No	Yes	Yes
Fibrin	No	Yes	Yes

**Table 5:** Comparative immunohistochemical markers for recent thrombus and agonal thrombus.

	Recent Thrombus	Agonal Thrombus
CD45	Yes	No
CD61	Yes	Yes
CD68	Yes	No
Von Willebrand factor	Yes	No
Fibrinogen	Yes	Yes
Fibrin	Yes	Yes

## Data Availability

Publicly available datasets were analyzed in this study. This data can be found here: https://docs.google.com/spreadsheets/d/1f80i3eht108WZY1HKl27nsrQHCpGtOGT/edit?usp=sharing&ouid=113085921623061867974&rtpof=true&sd=true (accessed on 1 August 2023).
